# A Rare Case of Central Nervous System Tuberculosis

**DOI:** 10.1155/2014/186030

**Published:** 2014-11-16

**Authors:** Ravish Parekh, Alexis Haftka, Ashleigh Porter

**Affiliations:** Henry Ford Hospital, Henry Ford Health System, 2799 West Grand Boulevard, K-7, Detroit, MI 48202, USA

## Abstract

Intracranial abscess is an extremely rare form of central nervous system (CNS) tuberculosis (TB). We describe a case of central nervous system tuberculous abscess in absence of human immunodeficiency virus (HIV) infection. A 82-year-old Middle Eastern male from Yemen was initially brought to the emergency room due to altered mental status and acute renal failure. Cross-sectional imaging revealed multiple ring enhancing lesions located in the left cerebellum and in bilateral frontal lobe as well as in the inferior parietal lobe on the left. The patient was placed on an empiric antibiotic regimen. Preliminary testing for infectious causes was negative. Chest radiography and CT of chest showed no positive findings. He was not on any immunosuppressive medications and human immunodeficiency virus (HIV) enzyme immunoassay (EIA) test was negative. A subsequent MRI one month later showed profound worsening of the lesions with increasing vasogenic edema and newly found mass effect impinging on the fourth ventricle. Brain biopsy showed focal exudative cerebellitis and inflamed granulation tissue consistent with formation of abscesses. The diagnosis of CNS TB was finally confirmed by positive acid-fast bacilli (AFB) cultures. The patient was started on standard tuberculosis therapy but expired due to renal failure and cardiac arrest.

## 1. Introduction

Central nervous system (CNS) tuberculosis (TB) is extremely rare without evidence of pulmonary tuberculosis [1]. In a large-scale epidemiological study of extra pulmonary tuberculosis in the United States, CNS involvement was noted in 5 to 10% of extra pulmonary tuberculosis cases [1]. Intracranial abscess is an extremely rare form of CNS TB. It occurs in only 4 to 8% of patients with CNS TB who do not have HIV infection [2]. There have been few case reports of CNS brain abscess in patients with HIV infection [3–5]. Due to low incidence of the disease in United States, the diagnosis is often delayed. We describe a case of central nervous system tuberculosis manifesting as brain abscesses in absence of HIV infection.

## 2. Case

**Figure 1:**
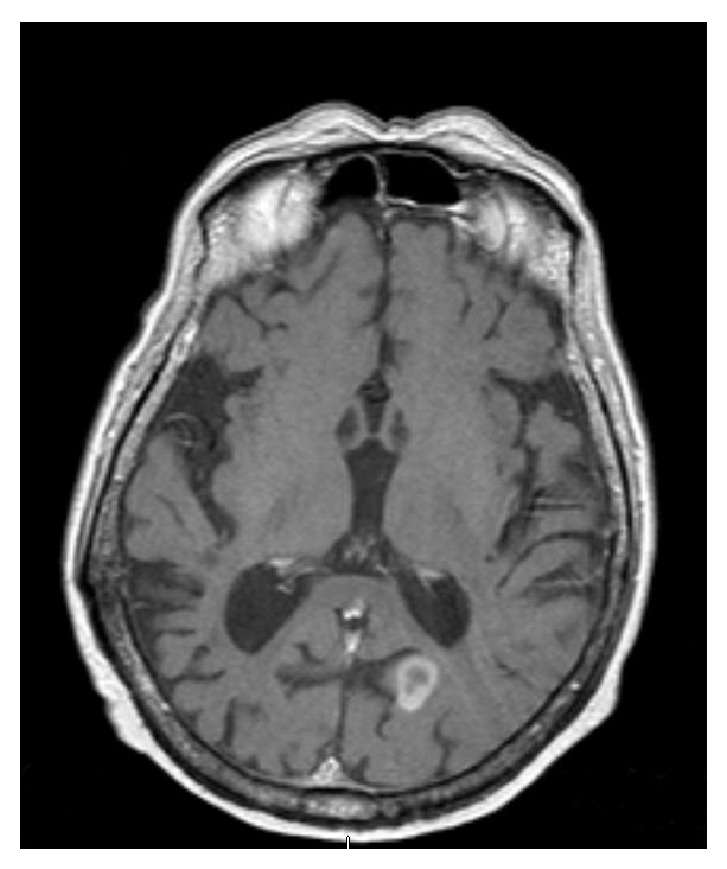
Magnetic resonance imaging of the brain showing ring enhancing lesion in the left parietal lobe, on initial presentation.

**Figure 2:**
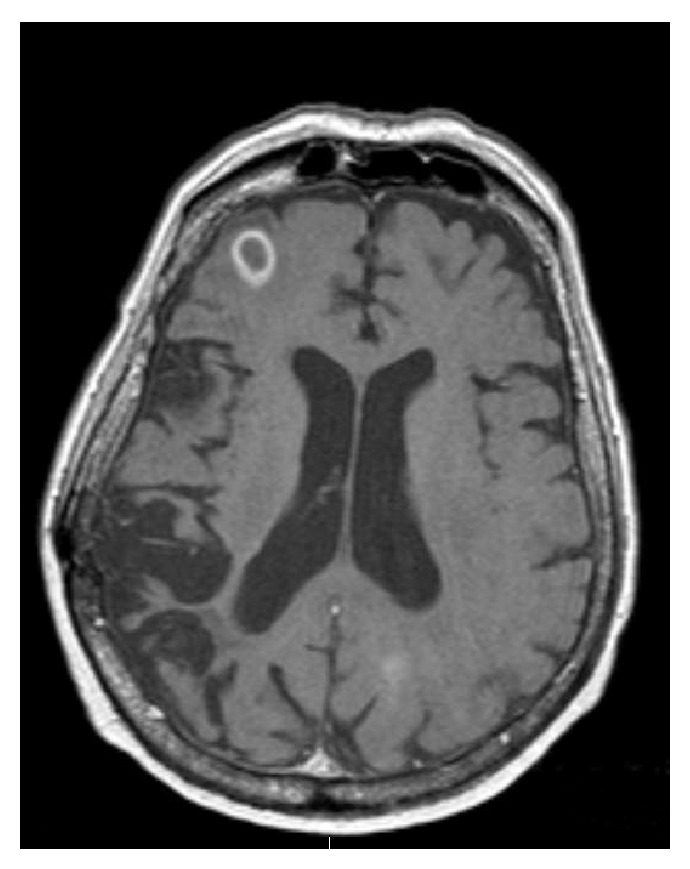
Magnetic resonance imaging of the brain showing ring enhancing lesion in the right frontal lobe, on initial presentation.

**Figure 3:**
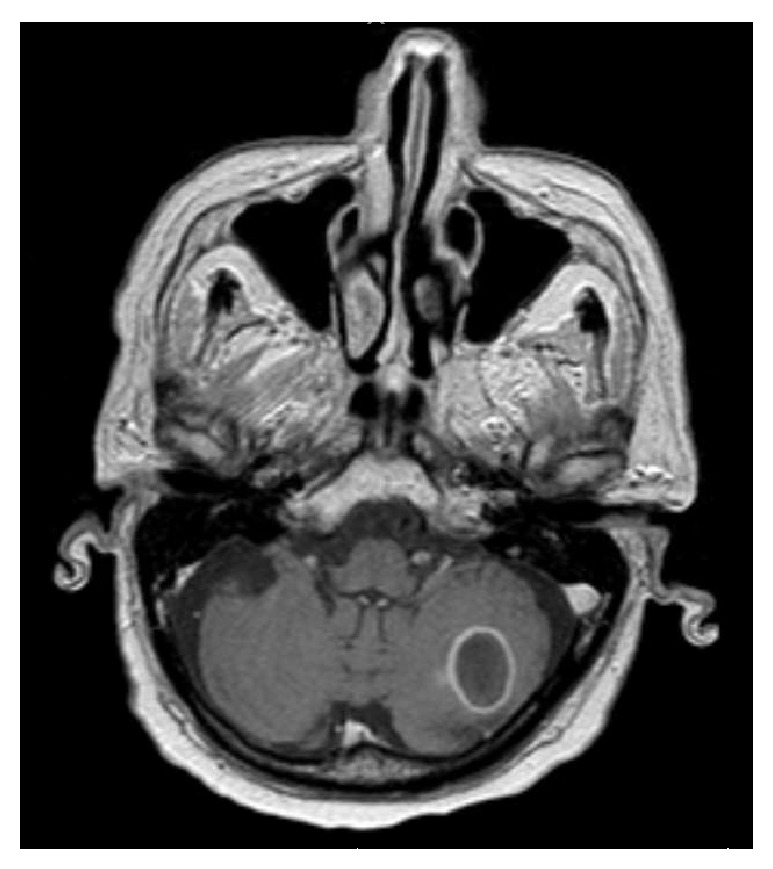
Magnetic resonance imaging of the brain showing ring enhancing lesion in the left cerebellum with mass effect on the fourth ventricle, on initial presentation.

An 82-year-old Middle Eastern male from Yemen with past medical history significant for atrial fibrillation (A-fib), type 2 diabetes mellitus, cryptogenic cirrhosis, and prostate cancer was initially brought to the emergency room due to altered mental status and acute renal failure. Computed tomography (CT) scan of the head and magnetic resonance imaging (MRI) of the brain revealed multiple ring enhancing lesions located in the left cerebellum, in bilateral frontal lobe, and in the inferior parietal lobe on the left (Figures 1, 2, and 3). The initial impression was infectious versus metastatic process as the patient had a recent history of prostate cancer; however, prostate specific antigen (PSA) levels were undetectable. The patient was placed on an empiric antibiotic regimen that included intravenous vancomycin, cefepime, and metronidazole based on Infectious Diseases Recommendation. Preliminary testing for infectious causes included an extensive workup including blood cultures, Fungitell, tuberculin skin (PPD) testing, bronchoalveolar lavage with culture, and interferon gamma release assay (Quantiferon) test for tuberculosis that was all negative. Chest radiography and CT of chest were also done which showed no positive findings. He was not on any immunosuppressive medications and human immunodeficiency virus (HIV) enzyme immunoassay (EIA) test was negative. While on intravenous antibiotics, patient initially had a marginal response with diminishing lesions and decreasing vasogenic edema on repeat MRI of brain after 2 weeks along with improving mental status.

**Figure 4:**
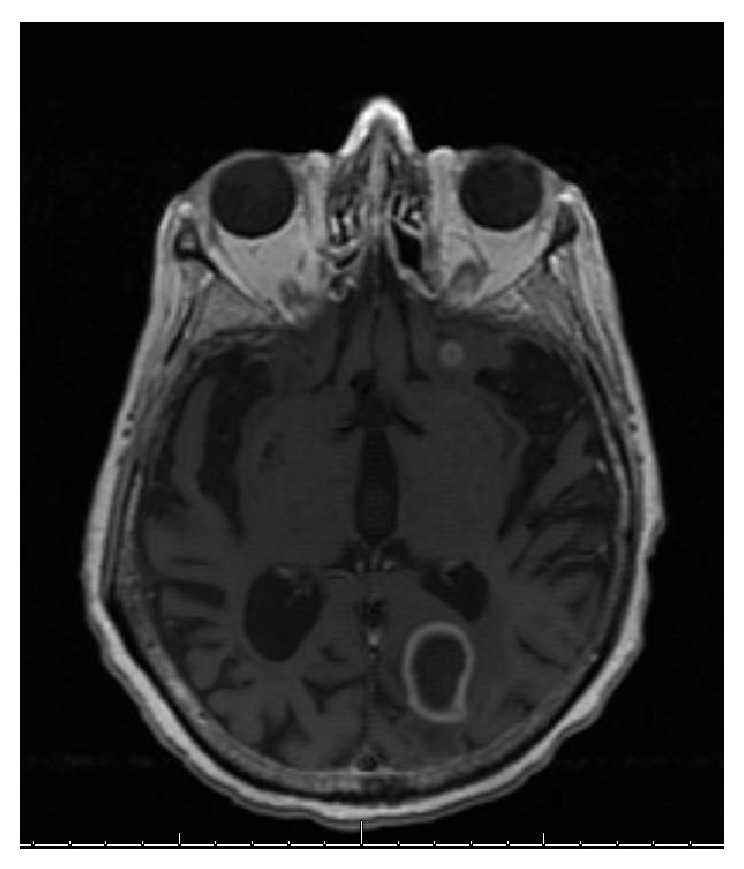
Magnetic resonance imaging of the brain showing ring enhancing lesion in the left parietal lobe, one month after initial presentation.

**Figure 5:**
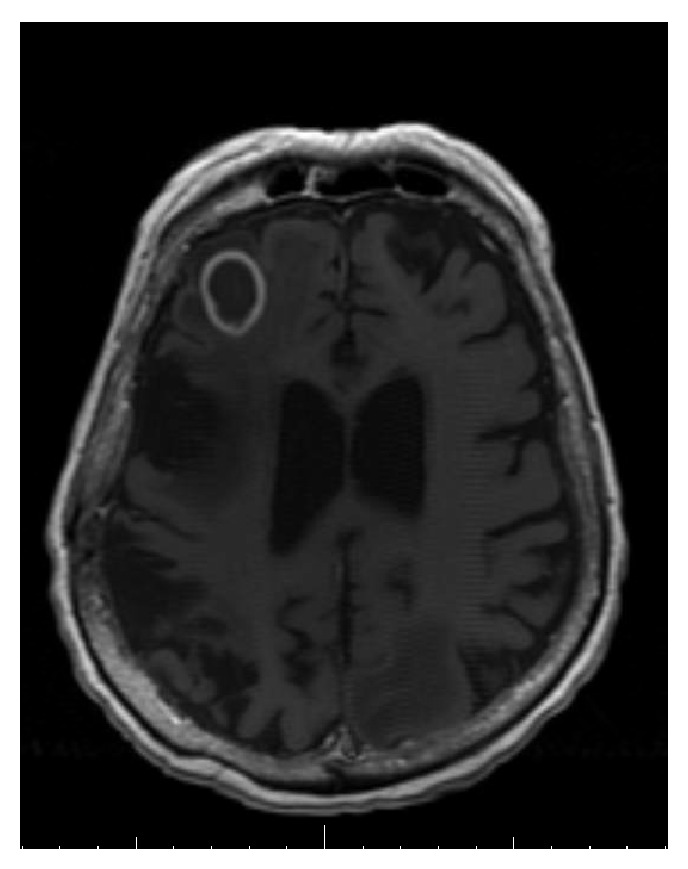
Magnetic resonance imaging of the brain showing ring enhancing lesion in the right frontal lobe, one month after initial presentation.

**Figure 6:**
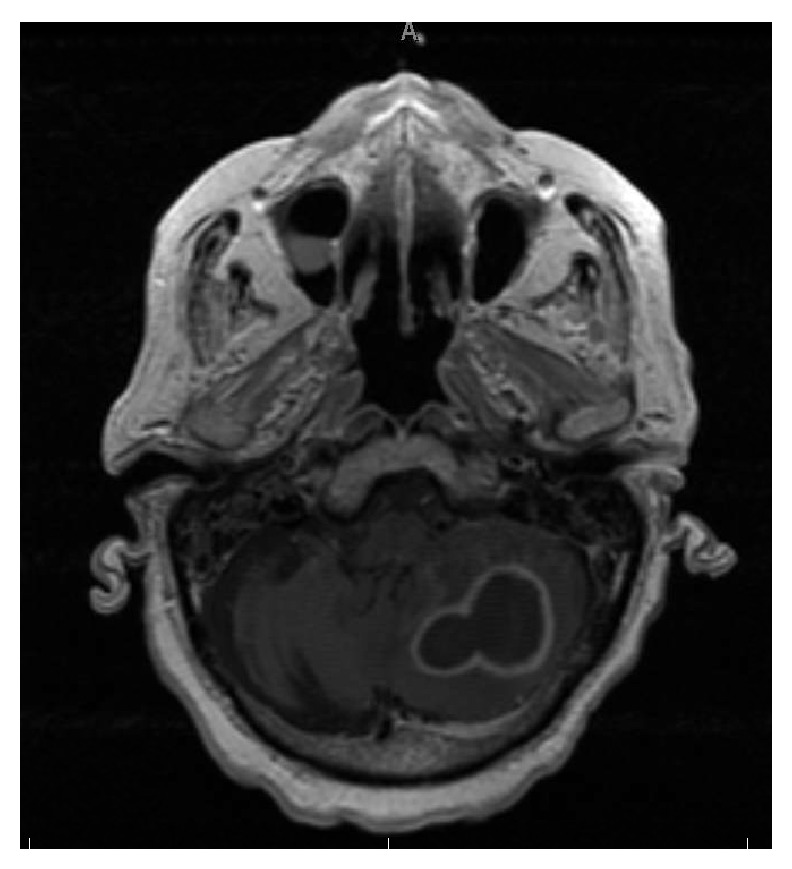
Magnetic resonance imaging of the brain showing ring enhancing lesion in the left cerebellum with mass effect on the fourth ventricle, one month after initial presentation.

Although the initial follow-up MRI showed a preliminary improvement in the size of the brain lesions, a subsequent MRI (Figures 4, 5, and 6) one month later showed profound worsening of the lesions with increasing vasogenic edema and newly found mass effect impinging on the fourth ventricle. The patient's course was further complicated by the development of generalized anasarca attributed to worsening renal function as well as poor nutritional status. The patient's mental status and overall health continued to decline and additionally developed A-fib with rapid ventricular response and respiratory distress. Furthermore, the patient was transferred to the intensive care unit with hypoxic respiratory failure due to increasing secretions and inability to protect his airway and was intubated for the remainder of his hospital stay. At this point, neurosurgery was consulted to biopsy the lesions in the brain. Due to patient's unstable hemodynamic status, brain biopsy was deferred until the patient stabilized three weeks later.

**Figure 7:**
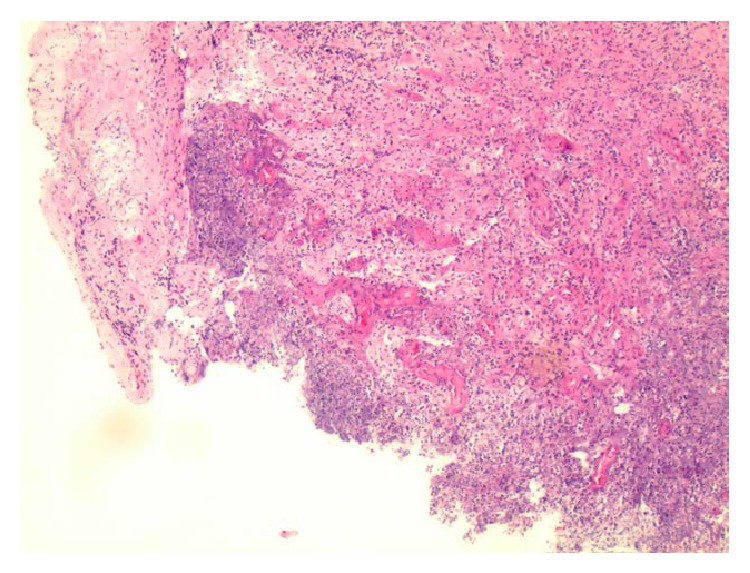
Brain biopsy of the ring enhancing lesion showing focal exudative inflammation with inflamed granulation tissue (Hematoxylin and Eosin stain, magnification ×40).

**Figure 8:**
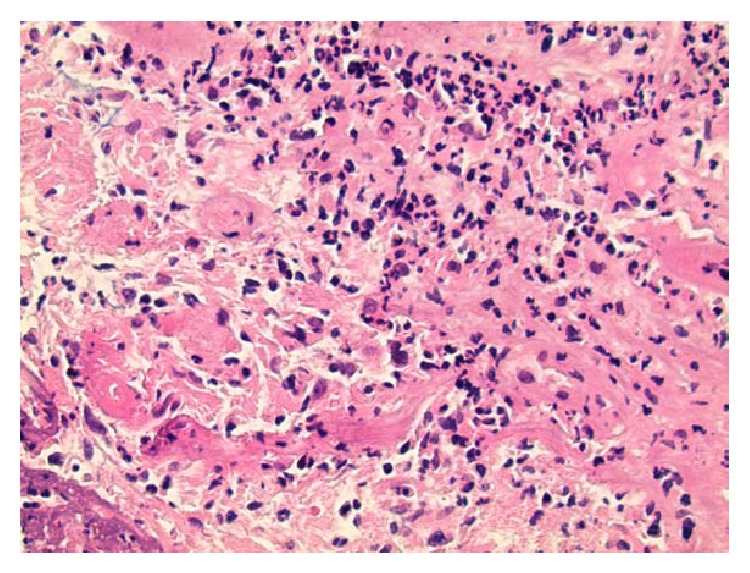
Brain biopsy of the ring enhancing lesion showing exudative inflammation consistent with an abscess (Hematoxylin and Eosin stain, magnification ×200).

**Figure 9:**
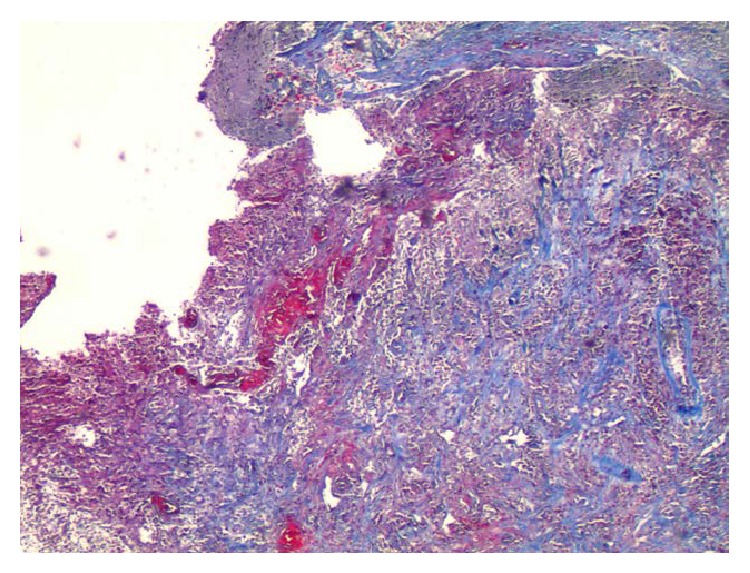
Biopsy of the brain lesion showing vascular proliferation and reticulin and collagen deposition within necrotic tissue (Trichrome stain, magnification ×40).

The biopsy of the brain lesions showed focal exudative cerebellitis and inflamed granulation tissue consistent with formation of abscesses (Figures 7, 8, and 9). The diagnosis of central nervous system (CNS) tuberculosis was finally confirmed by positive acid-fast bacilli (AFB) cultures. The following day the patient had a bronchial lavage, which was also AFB positive. The patient was started on standard tuberculosis therapy but unfortunately expired after 8 days of antibiotic treatment due to worsening renal failure and electrolyte abnormalities inducing cardiac arrest.

## 3. Discussion 

Tuberculosis (TB) is more frequently seen in endemic areas where there is a higher prevalence of tuberculosis. Over 95% of cases and deaths are in developing countries. In 2012, 8.6 million people fell ill with TB and 1.3 million died from TB [6]. In United States, the incidence of tuberculosis in 2012 was 3.2 percent [7]. CNS TB is extremely rare without evidence of pulmonary tuberculosis [1]. In a large-scale epidemiological study of extra pulmonary tuberculosis in the United States, CNS involvement was noted in 5 to 10% of extra pulmonary tuberculosis cases [1]. It is thought to be a postprimary result of systemic TB. CNS tuberculosis may present as a meningoencephalitis or spinal tuberculous arachnoiditis and rarely as CNS tuberculoma. In nonendemic areas, CNS TB usually affects the adult immigrants from areas of high prevalence, which is similar to our patient who had emigrated from Yemen [8]. Based on previous case reports the findings of CNS TB without systemic findings, such as pulmonary involvement, are very rare. Intracranial abscess is an extremely rare form of CNS TB. It occurs in only 4 to 8% of patients with CNS TB who do not have HIV infection [2]. There have been few case reports of CNS brain abscess in patients with HIV infection [3–5]. Our patient had a negative HIV test during the hospital stay and had no risk factors for HIV.

The source of brain abscess is thought to be hematogenous dissemination from the lungs like any other form of TB. It can occur from parenchymal tuberculous granuloma or via spread of tuberculous foci from meninges to the brain parenchyma [9]. Brain abscesses can be solitary or multiple and have a thicker wall than pyogenic abscess [2]. It is an encapsulated collection of pus with presence of tubercle bacilli that could be confirmed on culture results. In 1978, Whitener [2] devised criteria for the diagnosis of tuberculous brain abscess based on a review of 57 cases in world literature. Based on the criteria, there should be a macroscopic evidence of abscess formation within the brain parenchyma and it should be composed of vascular granulation tissue containing acute and chronic inflammatory cells. There should also be a proof of presence of tubercle bacilli demonstrated on culture or by positive acid-fast stain. Our patient did have presence of all the above criteria for the diagnosis of CNS brain abscess.

The imaging findings are predominantly multiloculated ring enhancing lesions with perilesional edema and mass effect [10]. Patients present with acutely deteriorating course and usual symptoms are headache, seizures, fever, focal neurological signs, or altered mental status [2]. Menon et al. [11] described a case series of tuberculous brain abscesses (TBA), where three patients were immunocompetent and only one patient had presented with altered mental status. They also described the use of in vitro proton magnetic resonance (MR) spectroscopy as a diagnostic modality for TBA. In another case series Cárdenas et al. [12] described six patients with TBA who were immunocompetent. All the patients on presentation had intracranial hypertension and focal neurological signs and symptoms on presentation. In comparison, our patient had no focal neurological signs on admission.

Studies have shown that PPD skin test and interferon gamma release assay (Quantiferon) have very low sensitivity in diagnosis of active TB [13, 14]. Our patient also had a negative Quantiferon test and PPD. Biopsy should only be done if the lesions are located in a safe location where there is a decreased probability of causing any injury to the brain.

Pyogenic brain abscess usually has a similar clinical presentation to TBA. They both usually have an acute presentation with similar signs and symptoms. Cerebrospinal fluid abnormalities are similar in both the diseases. The differentiating factor is usually a presence of extra cranial evidence of tuberculosis in patients with TBA [2]. In our patient, initially patient had no evidence of extra cranial TB. He also had negative AFB in sputum initially and no radiological evidence of pulmonary TB. Hence there was a diagnostic dilemma in our patient due to absence of any risk factors to have a suspicion for TB. He was initially being treated empirically for pyogenic brain abscesses that failed to show any clinical or radiological improvement in the brain lesions.

Treatment of tuberculous brain abscess should be approached with a combined medical and surgical management. Decreasing the size of space occupying lesion, relieving the raised intracranial pressure, and eradication of the tuberculous bacilli are the main goals of the treatment [12, 15–17]. Surgical literature has described stereotactic-guided aspiration, simple puncture, continuous drainage, and repeated aspiration through burr holes as the possible options [17]. However, early medical treatment with antituberculous therapy is extremely important if TBA is suspected. In Whitener's case series, forty percent (40%) of patients died in spite of antituberculous therapy [2]. First line therapy includes isoniazid, rifampin, ethambutol, streptomycin, and pyrazinamide. For the first two months of therapy the patient should receive 4 agents (isoniazid, rifampin, ethambutol, and streptomycin or pyrazinamide). After this two-month period, a patient should remain on therapy for additional 7–10 months of rifampin or INH. It is important to note that treatment can also cause a paradoxical phenomenon, which is believed to be a local hypersensitivity reaction to mycobacterial proteins released during the course of the treatment [18, 19].

Our patient was started on the intense regimen of isoniazid, rifampin, ethambutol, and pyrazinamide immediately after the biopsy results were reported, but unfortunately, due to the late diagnosis of CNS TB, our patient was unable to benefit from treatment. This case reemphasizes the need to have a high degree of suspicion for CNS TB in an appropriate clinical setting.
